# Influence-aware memory architectures for deep reinforcement learning in POMDPs

**DOI:** 10.1007/s00521-022-07691-7

**Published:** 2022-09-04

**Authors:** Miguel Suau, Jinke He, Elena Congeduti, Rolf A. N. Starre, Aleksander Czechowski, Frans A. Oliehoek

**Affiliations:** Intelligent Systems, Delft University of Technology, Delft, The Netherlands

**Keywords:** Partial observability, Reinforcement learning, Influence, Conditional independence, Recurrent neural networks

## Abstract

Due to its perceptual limitations, an agent may have too little information about the environment to act optimally. In such cases, it is important to keep track of the action-observation history to uncover hidden state information. Recent deep reinforcement learning methods use recurrent neural networks (RNN) to memorize past observations. However, these models are expensive to train and have convergence difficulties, especially when dealing with high dimensional data. In this paper, we propose *influence-aware memory*, a theoretically inspired memory architecture that alleviates the training difficulties by restricting the input of the recurrent layers to those variables that influence the hidden state information. Moreover, as opposed to standard RNNs, in which every piece of information used for estimating *Q* values is inevitably fed back into the network for the next prediction, our model allows information to flow without being necessarily stored in the RNN’s internal memory. Results indicate that, by letting the recurrent layers focus on a small fraction of the observation variables while processing the rest of the information with a feedforward neural network, we can outperform standard recurrent architectures both in training speed and policy performance. This approach also reduces runtime and obtains better scores than methods that stack multiple observations to remove partial observability.

## Introduction

It is not always guaranteed that an agent will have access to a full description of the environment to solve a particular task. In fact, most real-world problems are by nature partially observable. This type of problems can be modeled as *factored partially observable Markov decision processes (F-POMDP)* [15]. The model is an extension of the MDP framework [31] whereby, unlike in the original formulation, states are not assumed to be fully observable. This implies that the Markov property is no longer satisfied. That is, future observations do not solely depend on the most recent one. Moreover, in the factored formulation [4] states and observations are both defined by sets of variables, with the set observation variables being a subset of the set of state variables. This is because some of the variables that define the state space are hidden to the agent [22].

Most POMDP methods try to extract information from the full action-observation history (AOH) to disambiguate the hidden state variables. We argue however that, in many cases, memorizing all the observation variables is costly and requires unnecessary effort. Instead, we can exploit the structure of our problem and abstract away from our history those variables that have no direct influence on the hidden ones.

Previous work on *influence-based abstraction (IBA)* [28, 43] demonstrates that, in certain POMDPs, the non-Markovian dependencies in the transition and reward functions can be fully determined given a subset of variables in the history. Hence, the combination of this subset together with the current observation forms a Markov representation that is sufficient to compute the optimal policy. In this paper, we use these theoretical insights to propose a new memory model that tries to correct certain flaws in standard RNNs that limit their effectiveness when applied to reinforcement learning (RL). We identify two key features that make our model stand apart from the most widely used recurrent architectures, LSTMs [9] and GRUs [7]:

1. The input of the RNN is restricted to a subset of observation variables which, in principle, should contain sufficient information to estimate the hidden state.
2. There is a feedforward connection parallel to the recurrent layers, through which the information that is important for estimating Q values but that does not need to be memorized can flow.

Although these two features might be overlooked as minor modifications to the standard architectures, together, they provide a theoretically sound inductive bias that brings the structure of the model into line with the problem of hidden state. Moreover, as shown in our experiments, they have an important effect on convergence, learning speed, and final performance of the agents.

## Related work

*Partial observability* The problem of partial observability has been extensively studied in the past. The main bulk of the work, comes from the planning community where most solutions rely on forming a belief over the states of the environment using agent’s past observations [25, 30, 34]. Classic RL algorithms, on the other hand, cannot directly apply the above solution due to the lack of a fully specified transition model. Instead, they learn stochastic policies that rely only on the current observation [13, 18], or use a finite-sized history window to uncover hidden state [17, 21]. Even though the previous solutions do not scale to large and continuous state spaces, in the field of Deep RL the problem is most of the times either ignored, or naively overcome by stacking a window of past observations [23]. The paper by Steckelmacher et al [37] extends the options framework [39] by conditioning the option initiation policy on the previously-executed option, such that it can be applied to POMDPs that show certain hierarchical structure. Other approaches incorporate external memories [26] or use RNNs to keep track of the past history [1, 8, 14, 32]. Although this last solution scales better than observation-stacking, recurrent models are computationally expensive and often have convergence difficulties when working with high dimensions [15, 22]. A few works, have tried to aid the RNN by using auxiliary tasks like predicting game feature information [16] or image reconstruction [11]. We, on the other hand, recognize that the internal structure of standard RNNs might not always be appropriate and propose a new memory architecture that is better aligned with the RL problem.

*Attention* One of the variants of the memory architecture we propose implements a spatial attention mechanism [44] to provide the network with a layer of dynamic weights. This form of attention is different from the temporal attention mechanism that is used in seq2seq models [20, 42]. While the latter allows the RNN to condition on multiple past internal memories to make predictions, the spatial attention mechanism we use, is meant to filter out a fraction of the information that comes in with the observations. Attention mechanisms have recently been used in the context of Deep RL to facilitate the interpretation of the agent’s behavior [24, 40] or to tackle multi-agent problems [12]. Similar to our model, the architecture proposed by [36] also uses an attention mechanism to find the relevant information in the game screen and feed it into the RNN. However, their model misses the feedforward connection through which the information that is useful for predicting action values but that does not need to be stored in memory can flow (see Sect. 4.1 for more details).

## Background

The memory architecture presented in Sect. 4 builds on the F-POMDP framework, and the concept of *influence-based abstraction*. For the sake of completeness, we briefly introduce each of them here and refer interested readers to [15] and [27].

### Factored POMDPs

#### Definition 1

(F-POMDP) A factored POMDP (F-POMDP) is a tuple $\langle S,X,Y,A,T,R,O\rangle$ where *S* is the set of *k* state variables $S = \{S^1, ..., S^k\}$, such that every state $s \in \times _{i=1}^k S^i$ is a *k*-dimensional vector $s = \langle s^1, ..., s^k \rangle$, *X* is the set of *m* observation variables $X = \{X^1, ..., X^m \} \subseteq S$, such that every observation $o_t \in \times _{i=1}^m X^i$ is an *m*-dimensional vector $o = \langle x^1, ..., x^m \rangle$ with $m \le k$, *Y* is the set of *n* hidden state variables $Y = \{Y^1,...,Y^n\} \subseteq S$ with $n \le k$, $X \cup Y = S$, and $s_t = \langle o_t, y_t \rangle$, *A* is the set of actions, *T* is the transition function, $T(s_{t+1} \mid s_t,a_t)= \Pr (s_{t+1}\mid a_t,s_t)$, $R(s_t,a_t)$ is the reward function, $O(o_t \mid s_t)$ is the observation function.^1^

The task is to find the policy $\pi$ that maximizes the expected discounted sum of rewards [38]. Since the agent receives only a partial observation $o_t$ of the true state $s_t$, a policy that is based only on the most recent information can be arbitrarily bad [35]. In general, the agent is required to keep track of its past AOH to make the right action choices. Policies are therefore mappings from the full AOH $h_t = \langle o_0, a_0 ..., a_{t-1}, o_t\rangle$ to actions, $\pi (a_t \mid h_t)$.

### Memory

As mentioned in the previous section, ignoring the fact that the observations are not Markovian can lead to sub-optimal decisions. Therefore, most Deep RL methods that target partial observability use some form of memory to disambiguate hidden state. In our experiments we compare our method with the two techniques that are most widely used in practice.

*Frame stacking* This simple solution was popularized by the authors of the DQN paper [23], who successfully applied it to train agents on playing the Atari video games. Although the entire game screen is provided at every iteration, some of the games, contain moving sprites whose velocity cannot be measured using only the current frame. The solution they adopted was to provide the agent with a moving window of the past 4 observations. Of course, the practicality of this approach is limited to relatively small observation spaces and short history dependencies.

*Recurrent neural networks* A more scalable solution is to train an RNN on keeping track of the information by embedding the past AOH in its internal memory. However, standard recurrent neural networks, such as LSTMs [9] or GRUs [7] are known to be difficult to train and have convergence difficulties when dealing with high dimensions. The central argument of this paper is that these popular architectures, which were especially designed for a particular set of time series problems, (e.g. machine translation, speech recognition) are not the most suited for the RL task, as they fail to account for the structure many problems exhibit.

### Influence-based abstraction

The memory architecture we propose incorporates some of the theoretical insights developed by the framework of influence-based abstraction (IBA). Although we do not make strict use of the mathematical properties introduced below, we consider it important to include them here.

The fundamental idea of IBA is to build compact F-POMDP models in which hidden state variables are abstracted away by conditioning on the relevant parts of the agent’s AOH. Here, rather than simplifying the transition function, we use these insights to model the agent’s policy. Although according to the POMDP framework, optimal policies should condition on the full AOH, it turns out that, in most partially observable problems, not all previous information is strictly relevant.

**Fig. 1:**
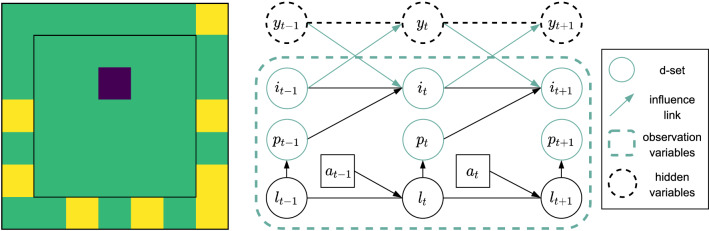
Left: A snapshot of the warehouse environment. The purple box represents a robot that needs to collect the yellow items that appear on the shelves located at the edges of the warehouse. Items disappear if they are not collected after 8 timesteps. Right: Dynamic Bayesian Network describing the environment dynamics. Edges represent conditional dependencies between variables. Variables within the green dashed box are visible to the agent. The hidden variables $y_t$ can only be inferred from the past AOH

*Example (Warehouse commissioning)* Figure 1 (left) shows a robot (purple) which needs to fetch the items (yellow) that appear with probability 0.05 on the shelves at the edges of the $7\times 7$ grid representing a warehouse. The robot receives a reward between (0, 1] every time it collects an item. The added difficulty of this task is that the robot is rewarded higher if it favors old over new item orders. Moreover, items disappear if they are not collected after 16 timesteps. Hence, the robot needs to maintain a time counter for each item and decide which one is best to go for.

The structure of the problem is represented by the dynamic Bayesian network (DBN) [5, 29] in Fig. 1 (right), where $l_t$ denotes the robot’s current location in the warehouse, and $i_t$ and $p_t$ are binary variables indicating if the item order is active and whether or not the robot is at the item pick-up location. The hidden state variable $y_t$ is the item’s time counter,^2^ to which the robot has no access, and upon which transitions and rewards depend. The robot can only infer the time counter based on past actions $a_t$ and observations $o_t = \langle l_t, p_t, i_t \rangle$. To do so, however, it does not need to remember the full AOH, but only whether or not a given item order was active at a particular timestep. More formally, inspecting the DBN, we see that $y_{t+1}$ is only indirectly influenced by the agent’s past location $l_{t-1}$ via $p_{t-1}$ and the item variable $i_{t}$. Therefore, we say that $y_{t+1}$ is conditionally independent of $l_{t-1}$ given $p_{t-1}$ and $i_t$,

1 $$\begin{aligned} \left( y_{t+1} \perp \!\!\!\!\perp l_{t-1} \mid p_{t-1}, i_t \right) \end{aligned}$$

Similarly,

2 $$\begin{aligned} \left( y_{t+1} \perp \!\!\!\!\perp a_{t-2} \mid p_{t-1}, i_t \right) \end{aligned}$$

The above means that in order to infer the hidden state variable *y* at any timestep it is sufficient to condition on the past values of *p* and *i*. The history of these two variables, highlighted in green in Fig. 1, constitutes the d-separating set (d-set).

#### Definition 2

(D-separating set) The d-separating set is a subset of variables $d_t$ in the agent’s AOH $h_t$, such that the hidden state variables $y_t$ and the remaining parts of the history $h_t \setminus d_t$ are conditionally independent given $d_t$: $\Pr (y_t\mid h_t) = Pr(y_t \mid d_t, h_t\setminus d_t) = Pr(y_t \mid d_t).$ This conditional independence can be tested using the notion of d-separation [3].

## Influence-aware memory

The properties outlined in the previous section, are not unique to the warehouse example. In fact, as we show in our experiments, it is often the case in partially observable problems that only a fraction of the observation variables influence the hidden state directly. This does not necessarily imply that the agent can completely ignore the rest of the information. In the warehouse example, the robot’s current location, despite being irrelevant for inferring hidden state, is in fact crucial for estimating action values.

The Bellman equation for the optimal action value function $Q^*$ of a POMDP can be expressed in terms of the history of actions and observations $h_t$ as

3 $$\begin{aligned} \begin{aligned} Q^*(h_t, a_t)&= R(h_t,a_t) + \sum _{o_{t+1}} \Pr (o_{t+1}\mid h_t,a_t) \max _{a_{t+1}} Q^*(h_{t+1},a_{t+1}), \end{aligned} \end{aligned}$$

with

4 $$\begin{aligned} \Pr (o_{t+1}\mid h_t,a_t) = \sum _{o_{t+1},s_{t+1}, y_t}O(o_{t+1} \mid s_{t+1})T(s_{t+1}\mid o_t,y_t, a_t) Pr(y_t\mid h_t) \end{aligned}$$

where $R(h_t, a_t) = \sum _{s_t} \Pr (s_t\mid h_t)R(s_t,a_t)$ is the expected immediate reward at time *t* over the set of possible states $s_t$ given a particular history $h_t$.

According to IBA, we can replace the dependence on the full history of actions and observations $h_t$ by a dependence on the d-set $d_t$ (Definition 2),

5 $$\begin{aligned} \begin{aligned} Q^*(\langle d_t, o_t \rangle , a_t) = R(\langle d_t, o_t \rangle ,a_t)&\\ + \sum _{o_{t+1},s_{t+1}, y_t} O(o_{t+1}\mid s_{t+1})T(s_{t+1}&\mid \langle y_t, o_t \rangle ,a_t)Pr(y_t\mid d_t) \max _{a_{t+1}} Q^*(\langle d_{t+1}, o_{t+1} \rangle , a_{t+1}), \end{aligned} \end{aligned}$$

and $d_{t+1} \triangleq \langle d_t, D(o_{t+1})\rangle$, where $D(\cdot )$ is the d-set selection operator, which chooses the variables in $o_{t+1}$ that are added to $d_{t+1}$. Note that, although $d_t$ contains enough information to estimate the hidden state variable $y_t$ (Equation 5 and Definition 2), $o_t$ is still needed to compute transitions *T* and rewards *R*. Hence, given the tuple $\langle d_t, o_t \rangle$ we can write

6 $$\begin{aligned} Q^*(h_t, a_t) = Q^*(\langle d_t, o_t \rangle , a_t), \end{aligned}$$

The upshot is that in most POMDPs the combination of $d_t$ and $o_t$ forms a Markov representation that the agent can use to find the optimal policy. Unfortunately, in the RL setting, we are normally not provided a fully specified DBN to determine the exact d-set. Nonetheless, in many problems like in our warehouse example it is not difficult to make an educated guess about the variables containing sufficient information to predict the hidden ones. The network architecture we present in the next section enables us to select beforehand what variables the agent should memorize. This is however not an prerequisite since, as we explain in Sect. 4.2, we can also force the RNN to find such variables by restricting its capacity.

### Influence-aware memory network

**Fig. 2:**
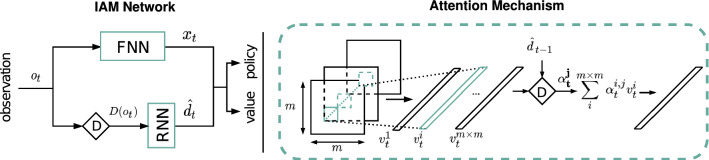
Left: Influence-aware Memory network architecture. IAM connects an FNN and RNN in parallel. While the FNN processes the entire observation vector, the RNN is fed only with the variables in $o_t$ that belong into $d_t$. Right: Diagram of one of the attention heads. Images are first processed by a CNN. The resulting feature map is decomposed into $m \times m$ vectors $v^i_t$ each of them describing the different region in the image. These vectors are fed into an attention module which computes a weight $\alpha ^i$ for each of them. The output of each attention head is the weighted average of these vectors

The Influence-aware Memory (IAM) architecture we propose is depicted in Fig. 2. The network encodes the ideas of IBA as inductive biases with the goal of being able to learn policies and value functions more effectively. Following from (6), our architecture implements two separate networks in parallel: an FNN, which processes the entire observation,

7 $$\begin{aligned} x_t = F_{\text {fnn}}(o_t), \end{aligned}$$

and an RNN, which receives only $D(o_t)$ and updates its internal state,

8 $$\begin{aligned} {\hat{d}}_{t} = F_{\text {rnn}}({\hat{d}}_{t-1},D(o_t)), \end{aligned}$$

where we use the notation ${\hat{d}}_t$ to indicate that the d-set is embedded in the RNN’s internal memory. The output of the FNN $x_t$ is then concatenated with ${\hat{d}}_t$ and passed through two separate linear layers which compute values $Q(\langle x_t,{\hat{d}}_t \rangle , a_t)$ and action probabilities $\pi (\langle x_t,{\hat{d}}_t \rangle , a_t)$.

**Fig. 3:**
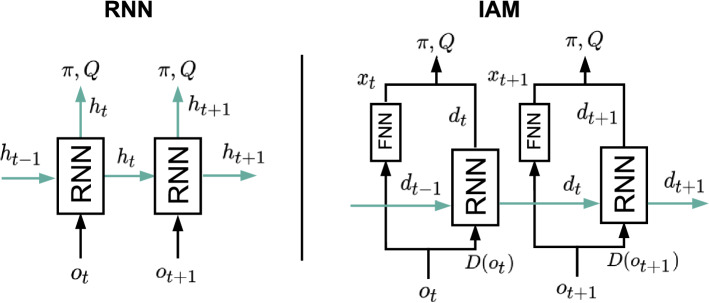
Information flow in standard RNNs (left) compared to IAM (right). The diagram on the left shows that the same vector $h_t$ that is used for estimating $\pi$ and *Q* is also part of the input for the next prediction (green arrows). On the other hand, in the IAM architecture there is another vector $x_t$ coming out from the FNN, which is only used for estimating $\pi$ and *Q* at time *t* and is not stored in memory. Hence, the RNN in IAM is free to include in ${\hat{d}}_t$ only the information that the agent needs to remember

*IAM vs. standard RNNs* We try to facilitate the task of the RNN by feeding only the information that, in principle, should be enough to uncover hidden state. This is only possible thanks to the parallel FNN channel, which serves as an extra gate through which the information that is useful for predicting action values but that does not need to be stored in memory can flow. This is in contrast to the standard recurrent architectures that are normally used in Deep RL (e.g. LSTM, GRU, etc.), which suffer from the fact that every piece of information that is used for estimating values is inevitably fed back into the network for the next prediction. Intuitively, standard RNNs face a conflict: they need to choose between ignoring those variables that are unnecessary for future predictions, risking worse *Q* estimates, or processing them at the expense of corrupting their internal memory with irrelevant details. Figure 3 illustrates this idea by comparing the information flow in both architectures.

Finally, since the recurrent layers in IAM are freed from the burden of having to remember irrelevant information, they can be dimensioned according to the memory needs of the problem at hand. This translates into networks that combine regular size FNNs together with small RNNs.

*Image data* If our agent receives images rather than feature vectors, we first preprocess the raw observations *o* with a CNN, $F_{\text {cnn}}(o_t) = \mathbf {v_t}$ and obtain $m \times m$ vectors *v* of size *N*, where *N* is the number of filters in the last convolutional layer and $m \times m$ the dimensions of the 2D output array of each filter (Fig. 2 right). Fortunately, since the convolution operator preserves the topology of the input, each of these vectors corresponds to a particular region of the input image. Thus, we can still use domain knowledge to choose which vectors should go into the RNN.

### Learning approximate d-sets

Having the FNN channel can help detach the RNN from the task of estimating the current *Q* values. However, without the d-set selection operator *D*, nothing prevents the information that does not need to be remembered from going through the RNN. Although, as we show in our first two experiments, it is often possible for the designer to guess what variables directly influence the hidden state information, it might not always be so straightforward. In such cases, rather than manually selecting the d-set, the agent will have to learn *D* from experience. In particular, we add a linear layer before the RNN, to act as information bottleneck [41] and filter out the irrelevant information:

9 $$\begin{aligned} {\hat{D}}_{A}(o_t) = A o_t \end{aligned}$$

where ${\hat{D}}$ indicates that the operator is learned rather than handcrafted and *A* is a matrix of weights of size $K \times N$, where *N* is the number of observation variables (the number of filters in the last convolutional layer when using images) and *K* is a hyperparameter that determines the dimensions of the output. The matrix A needs to be computed differently depending on the nature of the problem:

*Static d-sets* If the variables that must go into the d-set do not change from one timestep to another. That is, if *D* always needs to choose the same subset of observation variables, as occurs in the warehouse example, we just need a fixed matrix *A* to filter all observations in the same way. *A* can be implemented as a separate linear layer before the RNN or we can just directly reduce the size of the first recurrent layer.

*Dynamic d-sets* If, on the other hand, the variables that must go into the d-set do change from one timestep to another, we use a multi-head spatial attention mechanism [42, 44] to recompute the weights in every iteration. Thus we write $A_t$ to indicate that the weights can now adapt to $o_t$ and ${\hat{d}}_{t-1}$. The need for such dynamism can be easily understood by considering the Atari game of breakout. To be able to predict where the ball will be next, the agent does not need to memorize the whole set of pixels in the game screen, but only the ones containing the ball. A matrix $A_t$ that varies over time is needed because the location of these pixels differs in every observation. For each row *j* in $A_t$, each element $\alpha ^{i,j}_t$ is computed by a two-layer fully connected network that takes as input the corresponding element in the observation vector $o_i$ and ${\hat{d}}_{t-1}$, followed by a softmax operator. Figure 2 is a diagram of how each of the attention heads operates for the case of using as input the output of the CNN $\mathbf {v_t}$ instead of the observation vector $o_t$.

Note that the above solutions would not be able to filter out the information that is only useful for the current *Q* estimates without the parallel FNN connection (Fig. 3). It is also important to stress that these mechanisms are by no means guaranteed to find the optimal d-set. Nonetheless, as shown in our experiments, they constitute an effective inductive bias that facilitates the learning process.

## Experiments

We empirically evaluate the performance of our memory architecture on the warehouse example (Sect. 3), a traffic control task, the memory S11 environment from the gym-minigrid suite [6], and the *flickering* version of the Atari video games [8]. The goal of our experiments is to:

1. Evaluate whether our model improves over standard recurrent architectures. We compare learning performance, convergence and training time.
2. Show that our solution scales to high dimensional problems with continuous observation spaces.
3. Demonstrate the advantages of restricting the input to the RNN and compare the relative performance of learning vs. manually specifying the d-sets.
4. Analyze the impact of the architecture on the learned representations by inspecting the network hidden activations.

### Environments

Below is a brief description of the three domains on which we evaluate our model. Please refer to the "Appendix" for more details.

*Warehouse* This is the same task we describe in our example in Sect. 3.3. The observations are a combination of the agent’s location (one-hot encoded vector) and the 24 item binary variables. In the experiments where d-sets are manually selected, the RNN in IAM only receives the latter variables while the FNN processes the entire vector.

**Fig. 4:**
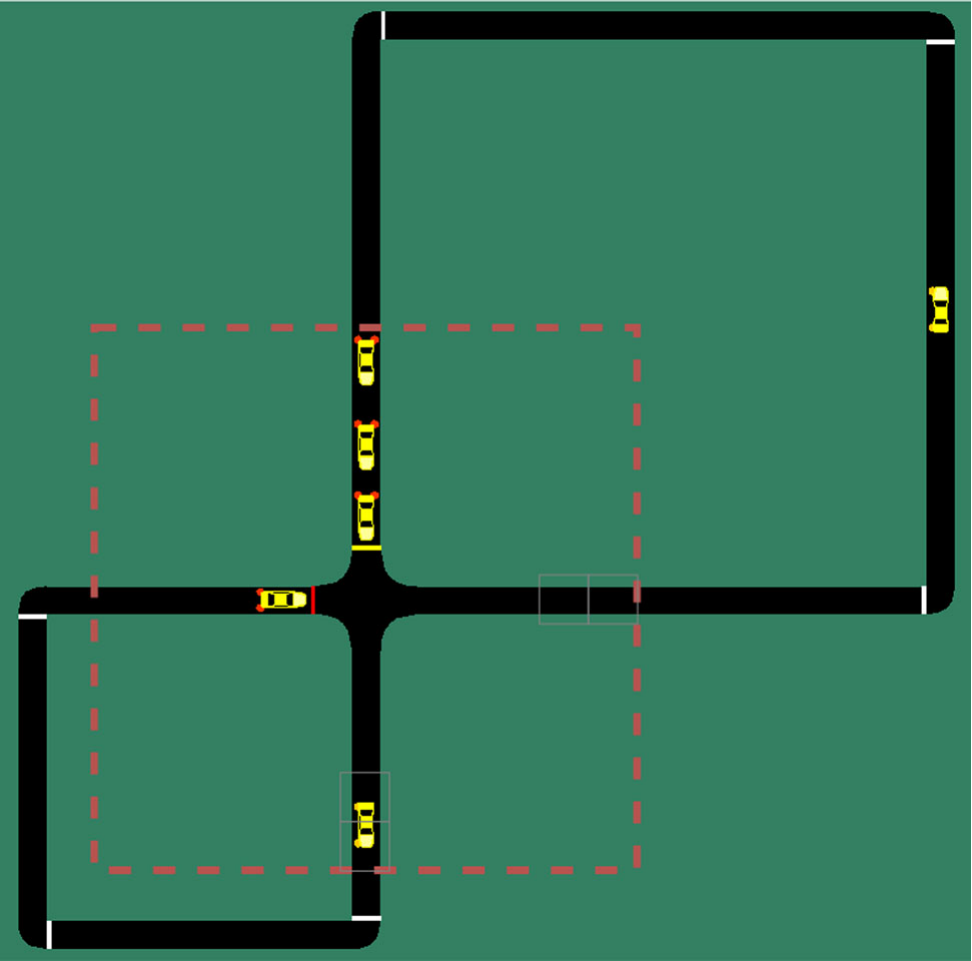
Traffic control environment. Cars are only visible when they enter the red box. The agent needs to anticipate the arrival of cars, and switch the lights before they enter the red box

*Traffic control* In this environment [19], the agent must optimize the traffic flow at the intersection in Fig. 4. The agent can take two different actions: either switching the traffic light on the top to green, which automatically turns the other to red, or vice versa. The observations are binary vectors that encode whether or not there is a car at a particular location. Cars are only visible when they enter the red box. There is a 6 seconds delay between the moment an action is taken and the time the lights actually switch. During this period the green light turns yellow, and no cars are allowed to cross the road.

Agents need to anticipate cars entering the red box and switch the lights in time for them to continue without stopping. This forces the recurrent models to remember the location and the time at which cars left the intersection and limits the performance of agents with no memory.^3^ In the experiments where d-sets are manually selected, the RNN in IAM receives the last two elements in each of the two vectors encoding the road segments (i.e. 4 bits in total). The location of these elements is indicated by the small grey boxes in Fig. 4. This information should be sufficient to infer hidden state.

*Gym-minigrid memory S11* The third environment is a high-dimensional version of the T-maze [1]. The environment is included in the gym-minigrid suite [6]. Here, the agent starts in a room where there is an object that it needs to memorize. Then, it has to go through a long corridor which ends in a split and choose one of the two pathways. The correct pathway depends on the object in the first room. A reward of $+1$ is given if the correct pathway is selected and 0 otherwise. To complicate things, the agent has limited vision and can only see objects that are within a $7\times 7$ grid from its own position. In the original implementation, the object in the first room is always at the same location. This makes it relatively easy to learn a fixed d-set operator ${\hat{D}}_A$ with static weights that can filter out all observation variables but the ones where the object is located. Hence, to make things harder, and in order to test the attention mechanism, we modified the environment so that the object is randomly placed at a different location in every episode.

*Flickering atari* In this version of the Atari video games [2] the observations are replaced by black frames with probability $p=0.5$. This adds uncertainty to the environment and makes it more difficult for the agent to keep track of moving elements. The modification was introduced by Hausknecht and Stone [8] to test their recurrent version of DQN (DRQN) and has become the standard benchmark for Deep RL in POMDPs [11, 45].

### Experimental setup

We compare IAM against two other network configurations: A model with no internal memory that uses frame stacking FNN, and two standard recurrent architectures GRU (warehouse, traffic, and memory S11 environments) and LSTM (Atari games). All four models are trained using PPO [33]. For a fair comparison, and in order to ensure that both types of memory have access to the same amount of information, the sequence length parameter in the recurrent models (i.e. number of time steps the network is unrolled when updating the model) is chosen to be equal to the number of frames that are fed into the FNN baseline. We evaluate the performance of our agents at different points during training by calculating the mean episodic return. The results are averaged over ten random seeds. A table containing the full list of hyperparameters used for each domain and for each of the three architectures, together with a detailed description of the tuning process is provided in the "Appendix".

### Learning performance and convergence

We first evaluate the performance of our model on the warehouse, traffic control, and memory S11 environments. Although the observation sizes are relatively small compared to most deep RL benchmarks (73, 30, and 49 variables respectively), the three tasks are quite demanding memory-wise. In the warehouse environment, the agent is required to remember for how long each of the items has been active. In the traffic domain, cars take 32 timesteps to reappear again in the red box when driving around the big loop (Fig. 4). Finally, in the Memory S11 the agent must remember the object it saw in the first room so that it can choose the right pathway at the end of the corridor.

**Fig. 5:**
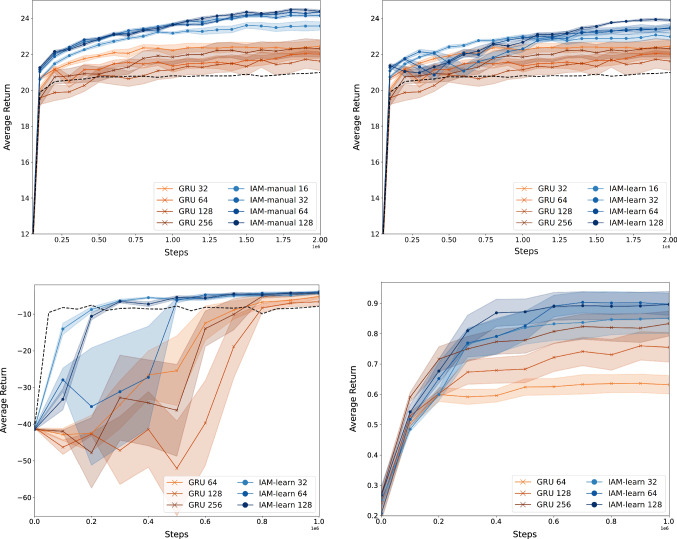
Average return and standard deviation during training of IAM and GRU for various recurrent layer sizes. Top left: IAM with manual d-set selection *D* on the warehouse environment. Top right: IAM with learned static d-set selection ${\hat{D}}_A$ on the warehouse environment. Bottom left: IAM with learned static d-set selection ${\hat{D}}_A$ on the traffic environment. Bottom right: IAM with learned dynamic d-set selection ${\hat{D}}_{A_t}$ on the Memory S11 environment. The dashed black lines are the learning curves of FNNs without memory

Figure 5, shows the learning curves of IAM and GRU in the three environments for various recurrent layer sizes. The IAM architecture outperforms the GRU baseline on all three environments both in terms of convergence and final performance. These results are strong evidence that the parallel feedforward channel in IAM is indeed helping overcome the convergence difficulties of GRUs (Sect. 4.1). Moreover, the size of the recurrent layers in IAM can be brought down to only ${\varvec{16}}$ neurons in the warehouse environment and ${\varvec{32}}$ neurons in the traffic and memory S11 environments while still outperforming both the GRU and the FNN baselines. This, of course, translates into a significant reduction in the total number of weights and thus computational speedups. A full summary of the average runtime for each architecture, along with a description of the computing infrastructure used is given in the "Appendix".

### Learning approximate d-sets

As explained in Sect. 4.2, if the optimal d-set is static, like in the warehouse and traffic environments, we might be able to learn ${\hat{D}}$ by simply restricting the size of the RNN. The two plots at the top of Fig. 5, show the difference in performance between manually selecting and learning the d-set on the warehouse domain.

The problem needs to be treated with a bit more care in cases where the variables that influence the hidden state change from one episode to another, as occurs in the Memory S11 environment. In such situations, just restricting the size of the RNN is not sufficient since the weights are static, and hence unable to settle for any particular subset of observation variables (Sect. 4.2). The plot at the bottom right of Fig. 5 shows the performance of IAM with a dynamic d-set selection layer ${\hat{D}}_{A_t}$ on the Memory S11 environment. As explained in Sect. 4.2, ${\hat{D}}_{A_t}$ is implemented by an attention mechanism. This layer makes the RNN module in IAM invariant to the location of the object, which translates into a significant performance gain with respect to the GRU baseline.

### High dimensional observation spaces

The advantage of IAM over LSTMs (GRUs) and FNNs becomes even more apparent as the dimensionality of the problem increases. Table 1 compares the average scores obtained in Flickering Atari by the FNN and LSTM baselines with those of IAM.^4^ Both IAM and LSTM receive only 1 frame. The sequence length parameter is set to 8 time steps for the two networks. The FNN model, on the other hand, receives the last 8 frames as input. The learning curves are shown in the "Appendix" together with the results obtained in the original games and the average runtime.

### Architecture analysis

*Decoding the agent’s internal memory*

**Table 1:** Average final score on the Flickering Atari games for each of the three network architectures and standard deviation

	FNN (8 frames)	LSTM	IAM
Breakout	$26.57 \pm 1.51$	$21.32 \pm 0.45$	$\mathbf {83.10} \pm \mathbf {5.29}$
Pong	$18.07 \pm 0.06$	$-20.25 \pm 0.03$	$\mathbf {20.07} \pm \mathbf {0.11}$
Space Invaders	$\mathbf {854.93} \pm \mathbf {11.64}$	$520.44 \pm 9.41$	$\mathbf {834.66} \pm \mathbf {21.23}$
Asteroids	$1393.75\pm 11.28$	$1424.87 \pm 5.23$	$\mathbf {2281.63} \pm \mathbf {63.92}$
MsPacman	$\mathbf {2388.03} \pm \mathbf {167.03}$	$1081.11 \pm 293.79$	$\mathbf {2326.04} \pm \mathbf {31.53}$

Bold numbers indicate the best results on each environment

**Fig. 6:**
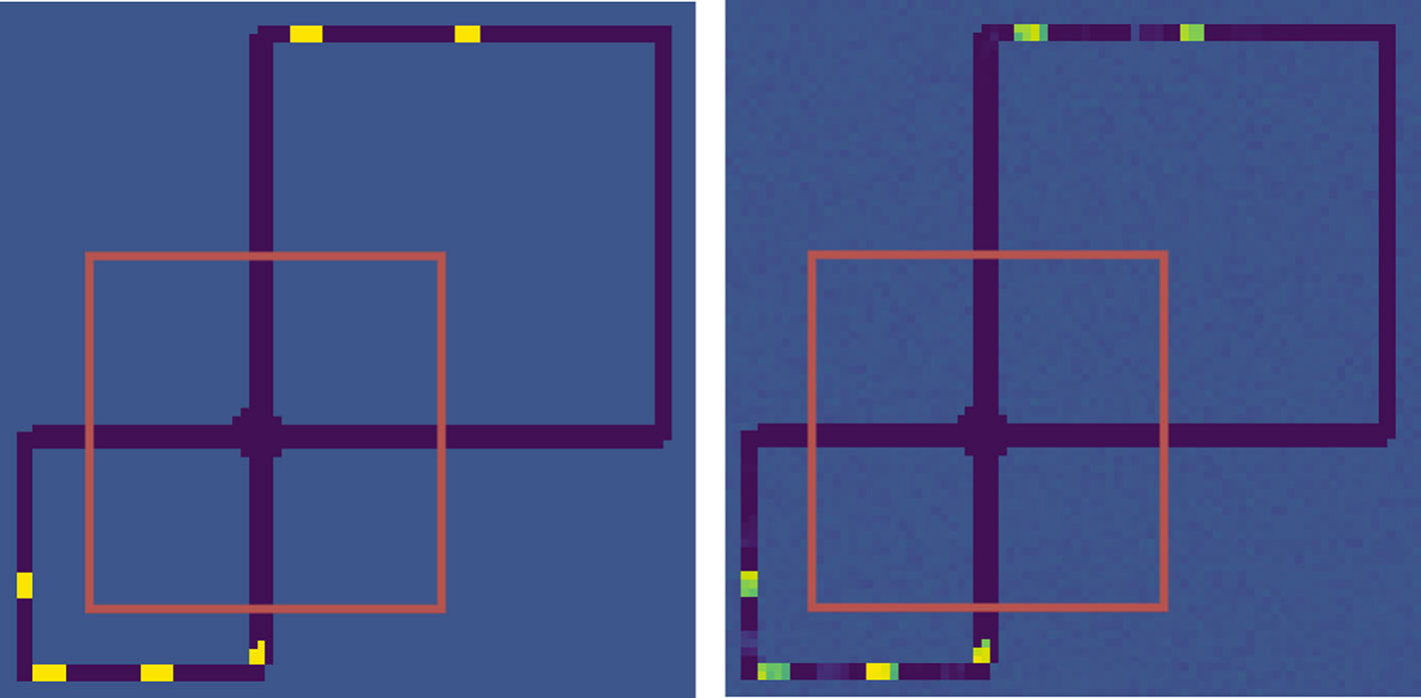
Example of a full simulator screen and the reconstruction made by the memory decoder (left). Although everything outside the red box is invisible to the agent, the decoder is able to make a fair reconstruction of the entire game screen based on the agent’s internal memory ${\hat{d}}$

We evaluated if the information stored in the agent’s internal memory after selecting the d-set and discarding the rest of the observation variables was sufficient to uncover hidden state. To do so, we trained a decoder on predicting the full game screen given the encoded observation $x_t$ and ${\hat{d}}_t$, using a dataset of images and hidden activations collected after training the policy. The image on the leftmost of Fig. 6 shows an example of the full game screen, from which the agent only receives the region delimited by the red box. The second image from the right shows the prediction made by the decoder. Note that although everything outside the red box is invisible to the agent, the decoder is able to make a fair reconstruction of the entire game screen based on the agent’s internal memory ${\hat{d}}$. This implies that IAM can capture the necessary information and remember how many cars left the intersection and when without being explicitly trained to do so.^5^

*Analysis of the hidden activations:*

**Fig. 7:**
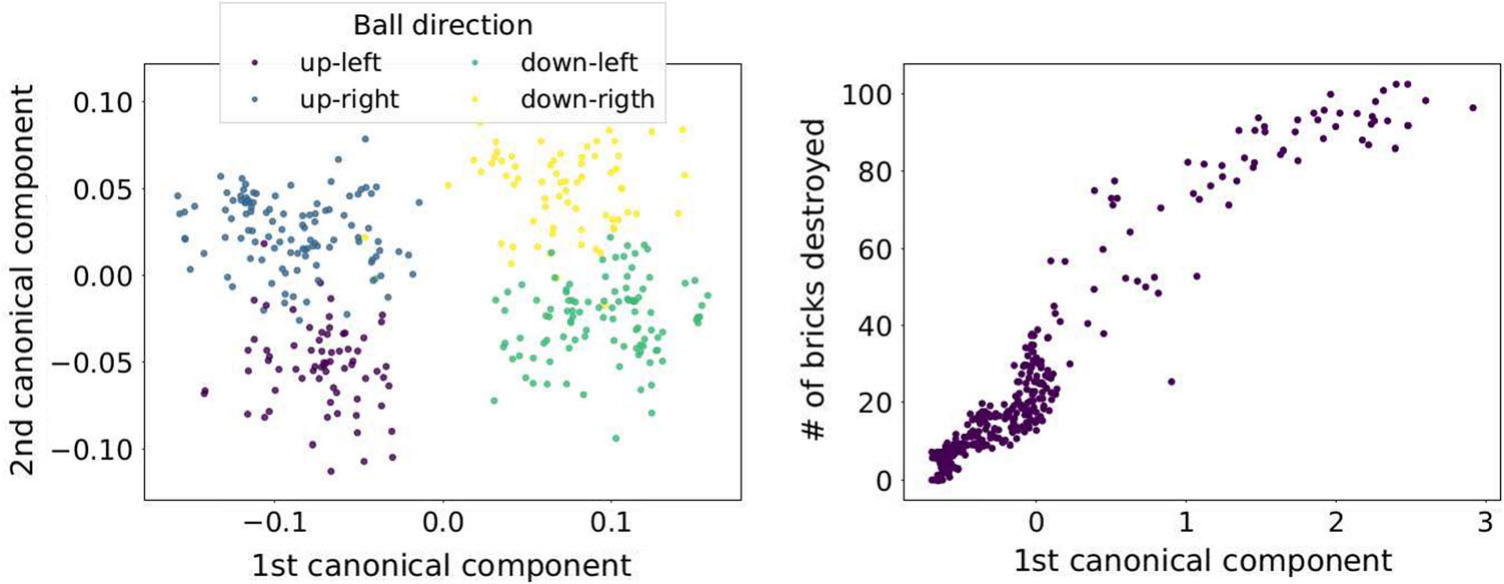
RNN’s internal memories ${\hat{d}}$ projected onto the two first canonical components, colors indicate the direction of the velocity vector (second from the right). FNN’s outputs *x* projected onto the first canonical component against the number of bricks destroyed (rightmost)

Finally, we used Canonical Correlation Analysis (CCA) [10] to measure the correlation between the network hidden activations when playing Breakout and two important game features: ball velocity and number of bricks destroyed. The projections of the hidden activations onto the space spanned by the canonical variates are depicted in the two plots on the right of Fig. 6. The scatter plot on the left shows four distinct clusters of hidden memories ${\hat{d}}_t$. Each of these clusters corresponds directly to one of the four possible directions of the velocity vector. The plot on the right, shows a clear uptrend. High values of the first canonical component of $x_t$ correspond to frames with many missing bricks. While the FNN is taking care of the information that does not need to be memorized (i.e. number of bricks destroyed) the RNN is focused on inferring hidden state variables (i.e. ball velocity). More details about this experiment are given in the "Appendix".

## Conclusion

The primary goal of this paper was to reconcile neural network design choices with the problem of partial observability. We studied the underlying properties of POMDPs and developed a new memory architecture that tries to decouple hidden state inference from value estimation. Influence-aware memory (IAM) connects an FNN and an RNN in parallel. This simple solution allows the RNN to focus on remembering just the essential pieces of information. This is not the case in other recurrent architectures. Gradients in LSTMs and GRUs need to reach a compromise between two, often competing, goals. On the one hand, they need to provide good *Q* estimates and on the other, they should remove from the internal memory everything that is irrelevant for future predictions. Our model enables the designer to select beforehand what variables the agent should memorize. This is however not an prerequisite since, as shown in our experiments, we can force the RNN to find such variables by restricting its capacity. We also investigated a solution for those problems in which the variables influencing the hidden state information differ from one observation to another. Our results suggest that while standard architectures have severe convergence difficulties, IAM can even outperform methods that stack multiple frames to remove partial observability. Finally, aside from the clear benefits in learning performance, our analysis of the network hidden activations suggests that the inductive bias introduced in our memory architecture enables the agent to choose what to remember.

## Figures and Tables

**Fig. 8:**
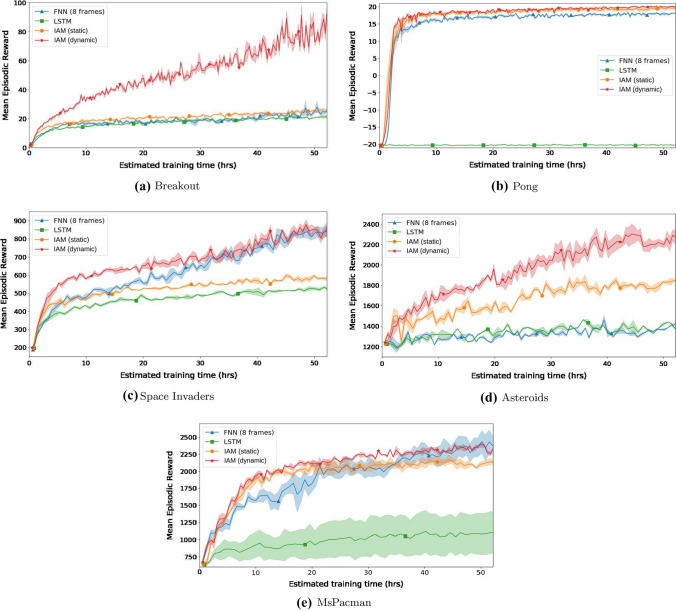
Average score per episode as a function of training time. Shaded areas show the standard deviation of the mean

**Fig. 9:**
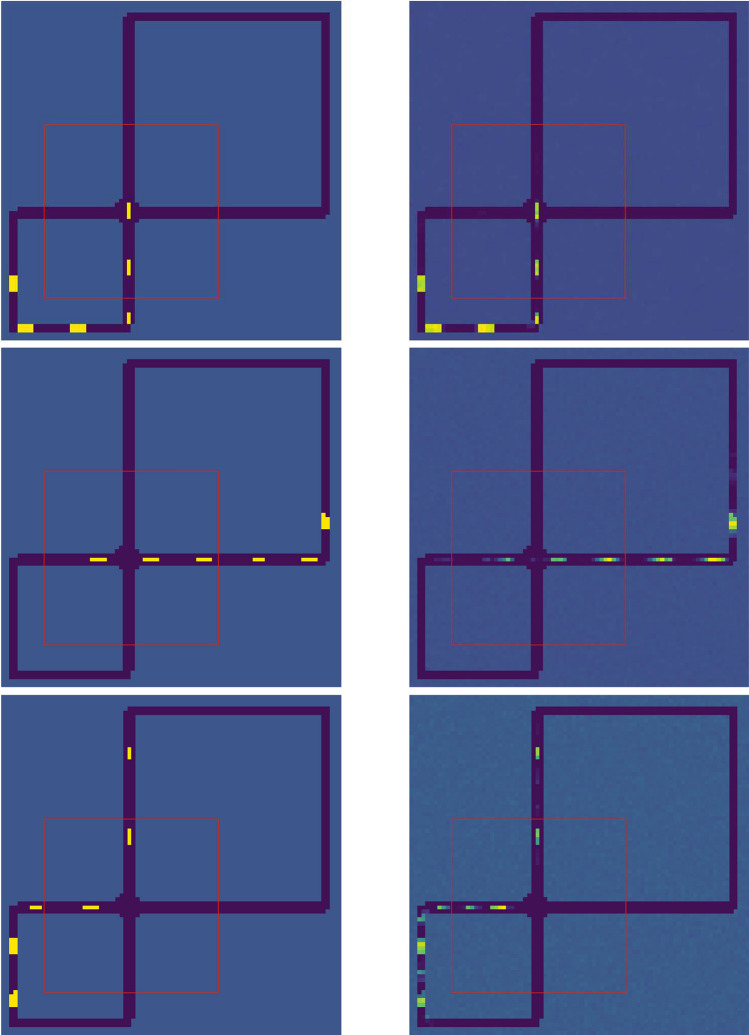
True state (left) and state predicted by the memory decoder (right)

**Fig. 10:**
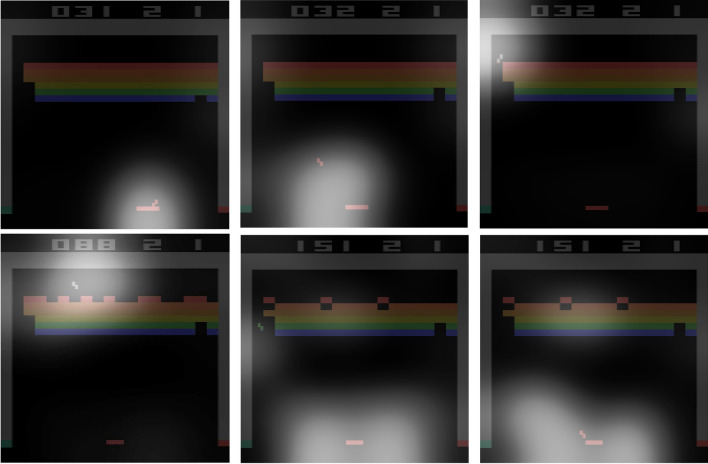
Breakout

**Fig. 11:**
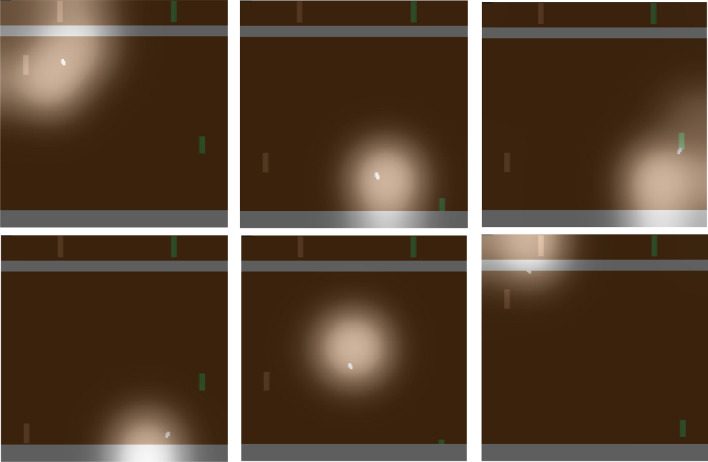
Pong

## Data Availability

The code used to run the experiments and generate the data for the current study is available in the Influence-Aware Memory repository https://github.com/INFLUENCEorg/influence-aware-memory.

## Appendix A: Implementation details

*Warehouse, traffic control, and memory S11* The FNN and GRU baselines that we used in the warehouse, traffic, and memory S11 environments, have two hidden layers. Only the first layer in the GRU baseline is recurrent. This seems to work much better than the reverse option (i.e. first layer feedforward and second layer recurrent). Moreover, to ensure a fair comparison, the size of the first layer in IAM is set equal to the size of the first layer in the FNN and the GRU baselines. That is, adding together the number neurons in the FNN and the GRU channels. For instance, if the first layer of the FNN and the GRU baselines is 640 neurons, a valid configuration for IAM would be 512 feedfoward and 128 recurrent neurons. Such that the FNN and the GRU combined also add up to 640. When doing observation-stacking the FNN baseline is provided the last 8 observations in the warehouse environment, 32 observations in the traffic environment, and 16 observations in the memory S11 environment. On the other hand, the gradients in the recurrent architectures (GRU and IAM) are backpropagated for 8, 32, and 16 timesteps (sequence length parameter). We did extensive testing to try to find the best possible network configuration for each model. These are reported in Table 2 for the warehouse environment, Table 3 for the traffic environment, and Table 4 for the Memory S11 environment. We tried different combinations of $\{128, 256, 512\}$ and $\{64, 128, 256\}$ for the size of the first and second layers respectively. As reported in Sect. 5.3 (Fig. 3), we also tried with $\{16, 32, 64, 128, 256\}$ neurons for the recurrent layers in GRU and IAM.

**Table 2:** Hyperparameters used for Warehouse Commissioning

Warehouse						
Model	FNN		GRU		IAM	
num frames	16		1		1	
time horizon	8		8		8	
seq length	–		16		16	
*FNN*	Layer 1	Layer 2	–	Layer 2	Layer 1	Layer 2
neurons	256	256	–	256	128	256
*RNN*	None		layer 1		layer 1	
rec neurons	–		256		128

**Table 3:** Hyperparameters used for Traffic Control

Traffic control						
Model	FNN		LSTM		IAM	
num frames	32		1		1	
seq length	–		32		32	
time horizon	32		32		32	
*FNN*	Layer 1	Layer 2	–	Layer 2	Layer 1	Layer 2
neurons	256	128	–	128	128	128
*RNN*	None		Layer 1		Layer 1	
rec neurons	–		256		128

**Table 4:** Hyperparameters used for Memory S11

Memory S11						
Model	FNN		GRU		IAM	
num frames	32		1		1	
time horizon	32		32		32	
seq length	–		32		32	
*FNN*	Layer 1	Layer 2	–	Layer 2	Layer 1	Layer 2
neurons	256	128	–	128	128	128
*RNN*	None		Layer 1		Layer 1	
rec neurons	–		256		128	
*Attention*	None		None		Layer 1	
neurons	–		–		32	
num heads	–		–		1	

*Flickering Atari* As explained in Sect. 4.1, when the observations are images, instead of feeding them directly into the FNN or the RNN, we first preprocess them with a CNN. The FNN and LSTM baselines use the same architecture reported by Mnih et al. [23] and Hausknecht and Stone [8] respectively. The IAM maintains the same CNN configuration, and then connects 128 feedforward and 128 recurrent neurons in parallel so that the total number of neurons (256) remains the same as in the FNN and LSTM baselines. Additionally, the attention mechanism, which computes $A_t$ for every $\langle o_t, {\hat{d}}_t\rangle$, consists of a single head with 256 neurons. The FNN baseline receives the last 8 frames as input, whereas LSTM and IAM only receive 1 frame. To update the network, gradients in the recurrent models are backpropagated for 8 timesteps. The network configurations used for Flickering Atari, are provided in Table 5. We used LSTM instead of GRU as the baseline for the flickering Atari games because this was the architecture used by Hausknecht and Stone [8].

**Table 5:** Hyperparameters used for Flickering Atari

Flickering Atari									
Model	FNN			LSTM			IAM		
num frames	8			1			1		
seq length	–			8			8		
time horizon	128			128			128		
CNN	Layer 1	Layer 2	Layer 3	Layer 1	Layer 2	Layer 3	Layer 1	Layer 2	Layer 3
filters	32	64	64	32	64	64	32	64	64
kernel size	8	4	3	8	4	3	8	4	3
strides	4	2	1	4	2	1	4	2	1
*FNN*	Layer 1			None			Layer 1		
neurons	512			–			256		
*RNN*	None			Layer 1			Layer 1		
rec neurons	–			512			256		
*Attention*	None			None			Layer 1		
neurons	–			–			256		
num heads	–			–			1		

As for the hyperparameters specific to PPO we used the same values reported by Schulman et al [33] for all three models and the three domains. These are shown in Table 6.

**Table 6:** PPO hyperparameters

learning rate	2.5e–4
discount $\gamma$	0.99
GAE $\lambda$	0.95
memory size	128
batch size	32
num. epoch	3
num. workers	8
entropy $\beta$	1.0e–2
clip $\epsilon$	0.1
value coeff. $c_1$	1

## B Runtime performance

Table 7 is an empirical analysis of the runtime performance of the three architectures. The values are the average wall-clock time in milliseconds of evaluating (forward pass) and updating (backward pass) the models. The FNN baseline receives a stack of 8, 32 and 8 observations in the Warehouse, Traffic Control and Flickering Atari environments, respectively. On the other hand, gradients in LSTM and IAM are backpropagated for 8, 32 and 8 timesteps when updating the networks in each of the three environments. An evaluation corresponds to a forward pass through the network. One update involves 3 epochs over a batch of 1024 experiences. The test was run on an Intel Xeon CPU E5-1620 v2.

**Table 7:** Runtime performance in milliseconds

	Warehouse		Traffic		Flickering Atari	
	Evaluation	Update	Evaluation	Update	Evaluation	Update
FNN (obs-stacking)	$1.03 \pm 0.41$	$81.96 \pm 1.29$	$0.90 \pm 0.16$	$63.21 \pm 0.71$	$3.82 \pm 0.53$	$215.95 \pm 7.54$
LSTM	$1.27 \pm 0.27$	$151.10 \pm 2.98$	$1.27 \pm 0.12$	$167.36 \pm 10.41$	$5.41 \pm 1.13$	$285.19 \pm 24.60$
IAM	$1.32 \pm 0.50$	$82.60 \pm 4.87$	$1.05 \pm 0.13$	$144.96 \pm 13.51$	$3.47 \pm 1.84$	$163.30 \pm 28.19$

## C Learning curves

Figure 8 shows the mean episodic reward comparison of the two baselines FNN and LSTM and the two versions of IAM, static and dynamic, as a function of training time on the *flickering* Atari games. Since we could not run all our experiments on the same machine, training time is estimated using the values provided in Table 7.

## D Results on the original Atari games

We also tested IAM on the original Atari games. Although the full game screen is visible at all times, some of the games contain moving elements whose speed and direction can not be measured from just a single frame. This means that the original games are also POMDPs [8]. In DQN [23] this issue is easily solved by stacking the last 4 frames and feeding them into the network. Our experiments show (Table 8), that even when frame-stacking seems to be the optimal solution, IAM can reach the same or even better performance of the FNN model while clearly outperforming the LSTM baseline.

**Table 8:** Average score per episode and standard deviation over the last 200K timesteps after 50 h of training on the original (non-flickering) Atari games

	FNN (4 frames)	LSTM	IAM (static)	IAM (dynamic)
Breakout	${\mathbf{326.54}} \pm {\mathbf{22.21}}$	$127.37 \pm 26.13$	$295.73 \pm 31.40$	${\mathbf{339.42 }}\pm {\mathbf{21.96}}$
Pong	${\mathbf{20.68 }}\pm {\mathbf{0.07}}$	$-20.26 \pm 0.04$	${\mathbf{20.73}} \pm {\mathbf{0.08}}$	${\mathbf{20.74 }}\pm {\mathbf{0.04}}$
Space Invaders	$975.39 \pm 70.06$	$1177.47 \pm 24.81$	${\mathbf{1326.63 }}\pm {\mathbf{53.59}}$	$868.23 \pm 37.34$
Asteroids	$2184.61\pm 62.83$	$1618.89 \pm 41.93$	$2057.21 \pm 109.65$	${\mathbf{2792.45 }}\pm {\mathbf{60.40}}$
MsPacman	${\mathbf{3859.91}} \pm {\mathbf{751.30}}$	$667.58 \pm 32.39$	${\mathbf{4016.78 }}\pm {\mathbf{273.11}}$	$3294.29 \pm 222.64$

The scores are also averaged over three trials. Bold numbers indicate the best results on each environment. Multiple results are highlighted when the differences are not statistically significant

## E Decoding the agent’s internal memory

The memory decoder consists of a two-layer fully connected FNN which takes as input the RNN’s internal memory ${\hat{d}}_{t-1}$ and the output $x_t$ of the FNN (see Fig. 3) and outputs a prediction for each of the pixels in the screen. The network is trained on a dataset containing screenshots and their corresponding network activations (${\hat{d}}_{t-1}$ and $x_t$), using the pixel-wise cross entropy loss. The dataset is collected after training the agent’s policy. The images are first transformed to grey-scale and then normalized to simplify the task. The results are shown in Fig. 9. The goal of this experiment was to confirm that although the RNN is only fed the last two elements of the binary vectors representing each lane, it can still uncover hidden state. It is important to point out that the agent is only trained to maximize the reward and has no explicit knowledge of the environment dynamics. A video of this experiment can be found at https://youtu.be/aDAjSzFC1bY.

## F Analysis of the hidden activations

We further analyze the information contained in ${\hat{d}}$ when playing Breakout (non-flickering) by looking at the activation patterns at different timesteps and their correlation with the ball velocity. The velocity vector *v* is computed by comparing the location of the ball at two subsequent frames. We use Canonical Correlation Analysis (CCA) [10] to obtain a lower dimensional representation of ${\hat{d}}$. In broad terms, CCA is a linear transformation that projects two sets of variables, in this case ${\hat{d}}$ and *v*, onto two subspaces that are highly correlated. This is done by iteratively finding linear combinations of ${\hat{d}}$ and *v*, known as canonical components, that maximize the correlation between them while being uncorrelated with the previous canonical components. We found that the first two canonical components of ${\hat{d}}$ are highly correlated with the two coordinates of the velocity vector ($\mathbf {0.98}$ and $\mathbf {0.90}$).

We also show that the FNN’s output *x*, on the other hand, contains information that does not need to be memorized but is relevant for predicting action values. We applied CCA to compare *x* with the number of bricks destroyed at each frame and obtained a correlation coefficient of $\mathbf {0.96}$. The projections of the ${\hat{d}}$ and *x* onto the space spanned by their corresponding cannonical components are shown in the two scatter plots on the left of Fig. 7 in the paper.

## G Attention maps

Here we include a collection of images which show the weights that the attention mechanism gives to the different regions of the game screen based on the agent’s internal memory ${\hat{d}}_{t-1}$ and the current observation $\hat{o_t}$. The network seems to focus on the regions that are important to memorize (Figs. 10, 11).

## Footnotes

1 Note that this formulation differs from the usual POMDP setting where the agent receives noisy observations of the true state, and where $O(o_t \mid s_t)$ is a probability distribution. Here, observations are a subset of the state variables, and hence $O(o_t \mid s_t)$ is a function that outputs a 1 if $o_t$ is a subset of $s_t$ and a 0 otherwise. Nonetheless, most POMDPs can be formulated as F-POMDPs by modelling the sources of partial observability as hidden state variables.

2 For simplicity, we only include a single item in the DBN in Fig. 1. The dynamics for all the other of the items in the warehouse are analogous.

3 Videos showing the results of the traffic control experiment can be found at https://youtube.com/playlist?list=PLbZouP4AED8DMxNb5oY8syc78-r6GOcDx.

4 We used LSTM instead of GRU as the baseline for the flickering Atari games because this was the architecture used by Hausknecht and Stone [8].

5 A video of this experiment where we use the decoder to reconstruct an entire episode can be found at https://youtu.be/aDAjSzFC1bY. More examples are provided in the "Appendix".
